# Health perspectives after intensive care unit-discharge: Insights from patient and family interviews

**DOI:** 10.1016/j.ijnsa.2025.100457

**Published:** 2025-11-15

**Authors:** Marisa Onrust, Ingeborg van der Meulen, Marie Louise Luttik, Wolter Paans, Peter H.J. van der Voort, Fredrike Blokzijl

**Affiliations:** a Department of Critical Care, University Medical Center Groningen, University of Groningen, the Netherlands; b Research Group Nursing Diagnostics, Hanze University of Applied Sciences, Groningen, the Netherlands; c Tias School for Business and Society, Tilburg University, Tilburg, the Netherlands; d Department of Cardiothoracic Surgery, University Medical Center Groningen, Groningen, the Netherlands

**Keywords:** Critical care, Family nursing, Follow-up studies, Postintensive care syndrome, Survivors, Qualitative research, Quality of life

## Abstract

**Background:**

The long-term consequences after an intensive care unit (ICU) hospitalization can be significant for both ICU survivors and their family members. Research in the past decades has shown that patients may develop new onset or worsened impairments in the physical, mental and cognitive domain and family members are known to experience psychological problems following ICU discharge. Furthermore, these impairments may affect daily functioning as well as family functioning.

**Aim:**

To gain insight into the way ICU survivors and their family members experience their health, three months after ICU discharge and to what extend this affects their roles and relationships within the family system.

**Design:**

An exploratory, qualitative study with in-depth interviewing.

**Setting(s):**

A large 38-bed ICU in a University Hospital in the Netherlands.

**Participants:**

Ten ICU-survivors and ten family members.

**Methods:**

ICU nurses performed in-depth interviews with ICU-survivors and family members, three months following discharge. Interviews were audio recorded and transcribed verbatim. Analysis was performed iteratively in accordance with the steps of inductive content analysis.

**Findings:**

Ten ICU-survivors and ten family members participated. We found four main themes: **personal autonomy, narrative reconstruction, relationship dynamics and empathetic concern,** which manifest different for ICU-survivors and family members, highlighting the distinct nature of their experiences. Physical recovery was a primary concern for ICU-survivors as well as family members, in order to regain personal autonomy. The different ICU-narratives of survivors and family members was hindering emotional recovery. Relationship dynamics occurred due to feelings of connection and disconnection intertwining, and empathetic concern was shaped by feelings of guilt and ambivalence.

**Conclusions:**

This study provides a deeper understanding of health perceptions of ICU-survivors and their family members, highlighting their contrasting experiences and the relational dynamics this can trigger. The findings of our study can be used to enhance the current approach of care after ICU discharge in any way, by actively involving the family system. Diagnostic, intervention, and outcome classification systems for nurses can be helpful in incorporating family-related aspects into the ICU context and aligning them with established family interventions, such as ‘the family health conversation’ and the ICU-diary.

**Registration:**

Not registered.

**What is already known**

• Health issues following ICU hospitalization due to critical illness have received more attention in society, healthcare, and research in recent years and knowledge about the impact on health related quality of life is increasing.
• The uncertainty and fear of potentially losing a loved one to critical illness contribute to the substantial impact that an ICU stay can have on family members. Research on this topic and knowledge about the long term consequences that may occur in family members has also grown.

Alt-text: Unlabelled box

**What this paper adds**

• This study describes how four shared themes emerged from the different perspectives of ICU survivors and their family members, and are reflected in four different subthemes for survivors and four subthemes for family members.
• The most important goal shared by both ICU survivors and family members in our study was to regain their personal autonomy. Nevertheless, the factors hindering this process were of different natures.
• The pattern of shared main themes with differing underlying causes —and, at times, consequences—highlights the value of a family-centered, holistic approach to recovery following ICU hospitalization.

Alt-text: Unlabelled box

## 1 Background

The long-term consequences of surviving critical illness including Intensive Care Unit (ICU) hospitalization are considerable for survivors and their family members. Symptoms reported by ICU-survivors include, among others, muscle weakness, fatigue, pain, cognitive impairments, depression, anxiety and Post-Traumatic Stress Disorder (Mäkinen et al., 2020; Müller et al., 2020). These symptoms are referred to as the Post Intensive Care Syndrome (Kosinski et al., 2020; Needham et al., 2012; Ramnarain et al., 2021). In family members, symptoms of anxiety and depression, Post-Traumatic Stress Disorder and complicated grief are reported and referred to as the Post Intensive Care Syndrome-Family (Davidson et al., 2012; Lobato, 2023; Shirasaki, 2024). The impact of PICS and PICS-F on daily living, health- and social status and Health Related Quality of Life can be disrupting (Gravante et al., 2024; Kerckhoffs et al., 2019). Increased health care consumption and loss of work or reduced working hours are described in previous studies (Gravante, 2024; Griffiths, 2013; Naaktgeboren, 2022).

Despite increasing knowledge about and research on the long-term consequences of surviving critical illness, knowledge about the consequences on family functioning remains scarce. Family Systems Theory is an approach that regards the family as a complex system with its own structures and patterns of interaction (Kerr and Bowen, 1988; MacKay et al., 2024). Family Systems Theory explores how family members’ behaviors and emotions are interconnected and influenced by the larger family dynamic. A dyad represents a fundamental subsystem within the family system, comprising two individuals who share a significant relational bond and whose interactions influence the broader family dynamics. The dyad serves as a critical relational unit, often shaping patterns of communication, emotional exchange and decision-making within the family. In 1990, Wright and Leahy introduced a systemic approach to nursing—Family Systems Nursing—based on Family Systems Theory. They described Family Systems Nursing as ‘focusing on the whole family as the unit of care,’ concentrating on both the individual and the family simultaneously with a focus on interaction and reciprocity (Shajani and Snell, 2023). Family Systems Nursing is the integration of systems theory, and family therapy theories’ into a nursing context (Wright and Leahy, 2009). Within the Family Systems Framework, two fundamental principles are always considered: 1) the patient relies on their family for recovery, and 2) illness (and care) within a family invariably affects everyone in the system (Kaakinen et al., 2014).

Given the potentially life-changing-event an ICU-hospitalization can be, it has impact on all individuals surrounding the patient. Partners of ICU-survivors, also referred to as dyads, likely experience the biggest impact on their daily life and have to cope with the consequences (Shirasaki, 2024). Dyadic coping models are developed during the last two decades, when a more systemic perspective on coping with stressful events was adopted (Falconier and Kuhn, 2019). The coping process, as elucidated by the dyadic coping models, situates itself within a relational framework wherein partners not only address their individual stress but also respond to the stressors of their partner. Within Family Systems Nursing, dyads are of great value in family health. Understanding of, and intervening in these dyadic relationships helps health care providers support both individual and family well-being. Family Systems Nursing emphasizes that health interventions should not only target the individual, but also consider the interconnected relationships within the family system (Landolt et al., 2023).

To effectively address the complexities of dyadic relationships and the interconnected family system, health care providers require structured frameworks that align with these relational dynamics. The NANDA (North American Nursing Diagnosis Association) (Herdman et al., 2024), NIC (Nursing Interventions Classification) (Wagner et al., 2023), and NOC (Nursing Outcomes Classification) (Moorhead et al., 2023) classification systems provide a framework for standardizing nursing practice, on the basis of which tailored interventions can be implemented to support both individual and family well-being. Several new items on family aspects are integrated in the latest versions of the NANDA, NIC and NOC classifications, as there is a growing recognition that family nursing becomes more and more important (Hagedoorn et al., 2021). Families often serve as the primary support system for ICU survivors. A deeper understanding of Post Intensive Care Syndrome and Post Intensive Care Syndrome-Family, and how these syndromes relate to dyadic coping and family functioning, is essential for healthcare providers—since such knowledge may help improve the support provided to both survivors and their families.

This study aims to explore how ICU-survivors and their family members experience their health three months post-discharge, and to what extent this influences their roles and relationships within the family system.

## 2 Methods

### 2.1 Study design, location and setting

We designed an exploratory qualitative study, utilizing semi-structured interviews as the primary method of data collection. This study was grounded in a constructivist paradigm in which reality is not fixed or objective, but shaped by social, cultural and personal context. The aim of research in the constructivist paradigm is to understand how people give meaning to their experiences. This approach allowed for an in-depth exploration of participants' perspectives and experiences within the context of the study aim. By utilizing semi-structured interviews, we aimed to facilitate open-ended discussions while also maintaining a degree of flexibility to probe further into emergent themes and insights.

ICU-survivors had been hospitalized to the ICU of the University Medical Center Groningen in the Netherlands. The ICU in this university center is a mixed ICU with 38 beds where approximately 3000 patients per year are admitted.

Family support interventions currently used in this ICU are not predefined in a fixed bundle, but are being provided on an ad hoc basis and largely depending on the individual nurse. Interventions comprise, among others, involvement of social work services, provision of an ICU diary, weekly family conversations, extended visiting hours, and unrestricted telephone access to the ICU.

### 2.2 Participants and recruitment

#### 2.2.1 Eligibility criteria

Inclusion criteria were: 1) age of 18 years or older and 2) an ICU Length of Stay (LoS) of 48 h or more. Family members could be a partner, brother, child or other important person identified as such by the ICU-survivor.

Exclusion criteria were: 1) communicational challenges, such as chronic mechanical ventilation or language barriers, 2) psychiatric history and 3) enrollment in another longitudinal study, to prevent overburdening of participants.

#### 2.2.2 Selection process

Initial screening was conducted by the first author, who is a research- and ICU nurse and a PhD student (MO). MO used the Electronic Patient Record to select ICU-survivors, who were subsequently approached by telephone, two to three months post-ICU-discharge. An experienced research nurse conducted the telephone call to inform survivors about the study and its purpose, and to obtain verbal informed consent. Additionally, permission to contact family members was requested. In case of consent, an information letter and informed consent form were send by mail. Written informed consent was obtained personally by the interviewer, prior to the start of the interviews.

### 2.3 Data collection

Patient characteristics (i.e. admitting diagnosis, length of ICU-stay) of ICU-survivors were retrieved from the Electronic Patient Record. Demographics of ICU-survivors and family members, such as age, gender, employment status and family composition, were collected during the interviews.

Semi-structured interviews were performed by four experienced ICU-nurses, who received interview training by one of the senior researchers and the second author (IvdM, assistant professor) prior to the interviews. The interviews were conducted at participants' homes. During the interviews with ICU-survivors, one nurse acted as the interviewer while the other acted as an observer, and this was reversed during the interviews with family members. During the interviews with the fourth survivor and the fourth family member, IvdM also acted as an observer, and during the interviews with the eighth survivor and the eighth family member, MO fulfilled this role. Fieldnotes were made by the observers and discussed afterwards with the aim to optimize the quality of the interviews and interview guide. Fieldnotes could include remarks about the interviewer’s attitude, silences that occurred or strategies to deepen the interview. The fieldnotes were not meant to obtain or analyze additional data.

During the interviews an interview guide with open-ended and non-leading questions was used, allowing participants to take as much space as they needed to discuss their experiences and perceptions. The interview guide was based on the Research ANd Development 36-item Health Survey (RAND-36) (Van der Zee and Sanderman, 2012), which is a validated and widely used questionnaire to measure experienced health and Health Related Quality of Life. The RAND-36 is grounded in a functional health perspective, which emphasizes individuals’ ability to perform daily activities rather than focusing solely on the presence or absence of disease. The questionnaire is based on a multidimensional concept of health (WHO, 1948) and on individuals' subjective perception of their own health (Ware and Sherbourn, 1992). The RAND-36 contains questions about physical functioning, social functioning, mental health, role limitation, vitality, pain, common health experience and changes in health. By using the RAND-36 questionnaire as the theoretical foundation, our study aimed to comprehensively explore the concept of health in its broadest sense, extending beyond the areas of Post Intensive Care Syndrome and Post Intensive Care Syndrome-Family. By employing this questionnaire as the foundation for the interviews, we anticipated that participants would be provided with ample opportunity to delve into nuanced aspects of their experienced health, including considerations of role performance and family functioning. Interviews with survivors and family members were guided by the same questions, given that the aim of the study was the same for both groups. Where appropriate, the questions were rephrased to suit the perspective of the family member. The interview guide is shown in Table 1, and table S1 in the supplementary file shows how the questions are mapped to the components of the RAND-36.

**Table 1:** Interview guide based on the RAND-36 questionnaire*.

1	How do you experience your current health ? (physical and psychological : feeling alive, energetic or tired)
2	What does health mean to you in your daily life ? (such as work, social life, relationships and hobbies)
3	In what way does your current healt affect your daily life ? (such as work, social life, relationships and hobbies)
4	In what way has your current health changed your daily life compared to before the ICU-hospitalization (of your relative)?
5	In what way has your current health changed your relationships with family members and/ or your partner ?
6	How do you deal with these changes ?
To encourage participants to eleborate on the topic: - Could you tell me more about that ? - How does that feel for you ? - What makes that so important for you ?	

⁎ Van der Zee KI, Sanderman R. Het meten van de algemene gezondheidstoestand met de RAND-36: Een handleiding. 2e herziene druk. [Measuring general health status with the RAND-36: A manual]. Groningen: Rijksuniversiteit Groningen, Research Institute SHARE; 2012.

### 2.4 Data analysis and trustworthiness

#### 2.4.1 Data collection and initial preparation of analysis

All interviews were audio-recorded and transcribed verbatim. Data collection and analysis alternated in an iterative process, ensuring the refinement of emerging themes (Hennink et al., 2020). A visualisation of the process is included in the supplementary file (figure S1). The first step in inductive content analysis according to Kyngäs (2020) is selecting the ‘units of analysis’, also referred to as ‘meaning units’. We chose to use sentences and words from the transcripts as our units of analysis, which enabled us to apply data extraction.

#### 2.4.2 Phase 1: initial data analysis and refining interview guide

The first round of interviews was conducted with four ICU-survivors and four family members. Data-reduction was performed by the interviewers (ICU nurses) and discussed with IvdM and MO. The interviewers identified open codes by reading through the raw data sentence by sentence and relating the sentence to the research question. The data from dyads were not examined differently than data from non-dyads. The transcripts were all analysed in an unmatched manner to ensure consistency in the analysis and maintain a standardized approach.

Data-grouping as the next step was also performed by the interviewers and again discussed with IvdM and MO. The coded data were grouped into sub-themes, again taking into account the focus of the study. Data-grouping was done by searching for differences and similarities in the abstracted data of the first step.

The third step in inductive content analysis is the formation of themes, which the interviewers conducted jointly by discussing the grouped data. The themes identified were subsequently used in their graduation theses.

Hereafter, based on the observations during the interviews and the initial data analysis, clarifications on operational level were made to the interview guide (e.g., clarifying 'socio-economic functioning') and extended questions on physical functioning were added to explore relationships.

#### 2.4.3 Phase 2: data analysis and confirming saturation

The second round of interviews was conducted with a new group of four ICU-survivors and four family members. Analysis was performed after these interviews by the interviewers with the help of MO, following the same steps as described above. To check if data saturation had been reached, two additional interviews (each with survivors and family members) were conducted. These interviews revealed no new codes and themes, confirming data saturation. A coding scheme with examples of meaning units, open codes, themes and main themes is included in the supplementary appendix (table S2).

#### 2.4.4 Phase 3: re-analysis, collaborative coding and refinement of themes

To ensure trustworthiness, the steps of data-reduction and data-grouping were performed once more on all transcripts by MO. The first, second and sixth transcripts of interviews with survivors and family members were analyzed independently by IvdM, FB (assistant professor and nurse specialist), and MO. Again were all the transcripts analyzed in an unmatched manner. Discrepancies in the open codes and grouped data were resolved through collaborative discussions, approached from a systemic perspective that emphasized the centrality of family and dyadic systems. The final step of formation of themes was performed in collaboration with MLL (associate professor) and WP (associate professor), who both have extensive experience with performing qualitative research, but are not familiar with ICU care. Discussing and refining themes culminated in a model visualizing themes and sub-themes (Fig. 2).

Finally, analyzed transcripts from ten ICU-survivors and ten family members confirmed no new insights likely.

#### 2.4.5 Validation of findings (Kyngäs, 2020, Kyngäs et al., 2020)

**Credibility** was achieved by investigator triangulation as multiple researchers were involved to reduce individual biases. Furthermore, the quotes that are used in this paper, were translated from Dutch by the University of Groningen Translation Agency, to ensure accuracy and preserve the meaning of the quotes

**Dependability** was achieved by maintaining a logbook (audit trail) for documenting decisions and reflections to account for researcher biases. Furthermore, we applied coding – recoding, peer-examination and collaborative discussions.

**Confirmability** was achieved by documenting reflections and observations collected during the interviews to ensure transparency in the research process.

**Authenticity** was achieved by selecting illustrative and relevant quotes from several different participants to support the findings.

**Transferability** was achieved by providing rich, thick descriptions for applicability in other contexts and by describing the sampling techniques, the inclusion criteria, and participants’ main characteristics.

We adhered the manuscript to the Standards for Reporting Qualitative Research (O’Brien et al., 2014) to comply to reporting standards.

#### 2.4.5.1 Ethical approval

This study has been submitted to the local Medical Ethical committee under the name ‘Follow-up Intensive Care Study (FICS)’. The Medical Ethical Committee approved the protocol for the FICS study (METc 2018/627) and waived the need for formal evaluation in accordance with the Dutch Law on Scientific Medical Research with Humans (Dutch Ministry of Health, Welfare and Sport, 1998). All participants received information on the study and gave written informed consent, prior to the interviews. To ensure anonymity, all data that may plausibly identify any of the participants were eliminated from the transcripts.

### 3 Findings

**Fig. 1:**
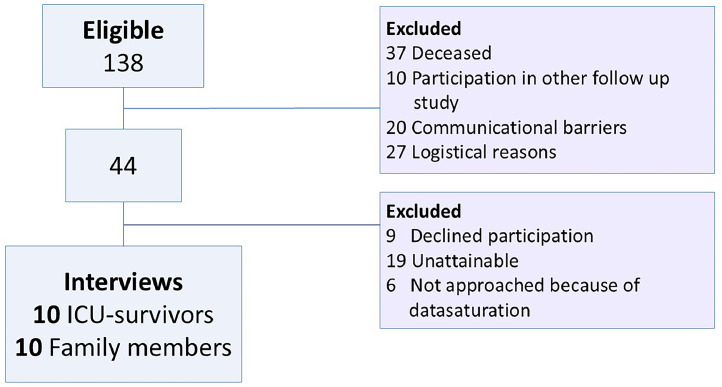
Flowchart of inclusion.

Recruitment took place over a nine-month period in which a total of 138 patients were discharged from the ICU and met the inclusion criteria. Of these, 37 died in the months following ICU-discharge and 57 were excluded based on the exclusion criteria (e.g. distance to the hospital and barriers in communication). Eventually, 44 survivors were suitable to approach for the study. Nine ICU-survivors did not want to participate and not all of the ICU-survivors could be reached by telephone. Finally, six potential participants were not approached, because data saturation had been achieved. The flowchart of the inclusion process is shown in Fig. 1.

#### 3.1 Participants

Ten ICU-survivors with a mean age of 63 (standard deviation [SD] 11) and ten family members with a mean age of 64 (SD 5,5) participated in the study. Of the ICU-survivors eight were male as were four of the family members. All family members were partners of ICU-survivors. Of three ICU-survivors, no family member participated and of three ICU-survivors, who did not want to participate themselves, the spouse did. The dyads who participated, were interviewed separately and independently from each other. On average, the interviews lasted one hour. Characteristics and demographics of the participants are shown in Table 2.

**Table 2:** Demographic characteristics of participants.

ICU survivors	Sex	Age	ICU LOS^1^ (days)	Delirium in ICU^2^	Employment status	Admitting diagnosis	Dyad^3^	Family members	Sex	Age	Employment status	Relationship to patient
P 1	M	58	13	Yes	Unemployed	Organ transplant		-	-	-	-	-
P 2	F	62	4	Yes	Housewife	Neurological		-	-	-	-	-
P 3	M	39	6	Yes	Self-employed	Organ transplant		-	-	-	-	-
P 4	M	73	4	Yes	Sick leave	Cardiac	Yes	**F 4**	F	69	Self-employed	Spouse
P 5	F	62	3	No	Sick leave	Organ transplant	Yes	**F 5**	M	64	Employed	Spouse
P 6	M	68	3	No	Retired	Trauma	Yes	**F 6**	F	60	Employed	Spouse
P 7	M	59	3	No	Sick leave	Cardiac	Yes	**F 7**	F	56	Employed	Spouse
P 8	M	61	11	Yes	Sick leave	Oncological	Yes	**F 8**	F	58	Employed	Spouse
P 9	M	70	5	Unknown	Retired	Cardiac	Yes	**F 9**	F	65	Housewife	Spouse
P 10	M	76	6	No	Retired	Respiratory	Yes	**F 10**	F	72	Retired	Spouse
	*F*	*69*	*6*	*Unknown*	-	Neurological		**F 1**	M	69	Retired	Spouse
	*F*	*60*	*3*	*No*	-	Cardiac		**F 2**	M	61	Employed	Spouse
	*F*	*62*	*16*	*No*	-	Respiratory		**F 3**	M	70	Retired	Spouse
Mean (SD)		63 (9,2)	6,4 (4,3)							64 (5,5)		

1 LOS: Length of Stay.

2 Delirium in ICU was extracted from the patients’ medical record.

3 Seven dyads participated in the interviews who were interviewed separately and independently from each other.

#### 3.2 Themes

**Fig. 2:**
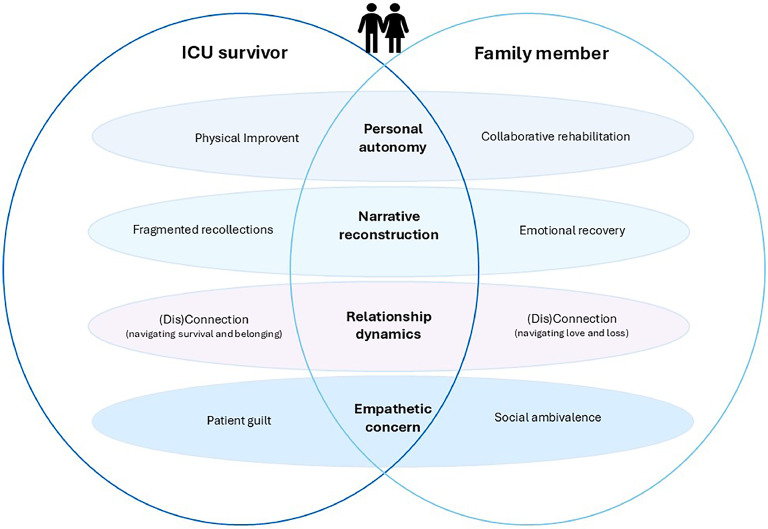
Visualization of the themes.

The analysis revealed four main themes addressing the research aim; **personal autonomy, narrative reconstruction, relationship dynamics and empathetic concern**. Within these themes we identified four subthemes in ICU-survivors as well as in family members. A visualization of the themes and subthemes is shown in Fig. 2.

#### 3.3 Main theme 1: personal autonomy

Personal autonomy encompasses the ability to think independently, make choices, and act upon them, reflecting one's own values and beliefs without external interference. Both ICU-survivors and family members reflected on striving to regain their personal autonomy. ICU-survivors showed an individual focus on physical improvement, whereas family members showed a collaborative focus.

##### 3.3.1 ICU-survivors: physical improvement

The subtheme physical improvement refers to the process of rehabilitation after critical illness, which is necessary for ICU-survivors to become an autonomic person again. Although not all of the ICU-survivors were still undergoing rehabilitation with a physiotherapist and designed interventions, their primary daily activities appeared to be focused on recovery and physical improvement. They used their current activity level as a measure of recovery.

*‘I’m training, because I still have very little energy and very little strength. I have a spinning bike here at home, so I’m also using that again. And I try to do some jobs around the house from time to time, but all of that takes a lot more energy than before. I want to start doing it again, but it takes time.’(P6)*

Survivors often compared their current Health Related Quality of Life to their pre-ICU situation. Notably, survivors following a solid organ transplant, rated their current Health Related Quality of Life higher than before the ICU stay, whereas survivors who were urgently hospitalized experienced their Health Related Quality of Life to be lower than before the ICU stay. The physical wellbeing of survivors who underwent an organ transplant had improved enormously.

*‘True, I can really notice the progress. Before the surgery, I couldn’t even make it to the end of the street. The heart was worn out and I was worn out myself as well.’ (P3)*

##### 3.3.2 Family members: collaborative rehabilitation

Family members tried to collaborate with their relatives by organizing joint activities that could simultaneously serve as a training or exercise for the ICU-survivor. This showed that, for most of the family members, their lives still revolved around the ICU-survivor’s rehabilitation, because their role as informal caregiver, and consequently the physical and cognitive limitations of their relative, often prevented them from continuing their own lives.

*‘When I leave the house, he comes with me. I think he should walk! (laughs) He’s relying on me a lot, but in the end he needs to slowly regain his self-confidence again.’ (F9)*

Some family members indicated that newly developed impairments, such as difficulty concentrating and becoming easily overstimulated, prevented them from fully regaining their personal autonomy but also motivated them to support their partners’ rehabilitation.

*‘I’m attending even fewer meetings. Before, I used to like those. Last week, I attended a meeting again for the first time and, to be honest, it was too much. That’s a big difference compared to before my husband's accident. I have more trouble focussing and I’m more forgetful.’* (F6)

#### 3.4 Main theme 2: narrative reconstruction

Discrepancies in how individuals perceive or interpret the same event, often arise from differences in their experiences, beliefs, and cognitive processes. ICU-survivors and their family members recount the same experience from contrasting perspectives. Factors such as sedation, which can lead to fragmented memories for ICU-survivors, and the intermittent presence of family members contribute to the misalignment of their ICU narratives.

##### 3.4.1 ICU-survivors: fragmented recollections

Most ICU-survivors could not remember anything coherent of the ICU-experience, but some of them had memories of distorted and frightening dreams.

*‘I remember a string of nightmares. I couldn’t get away from them and afterwards, when I woke up, I was completely lost. I still think about that.’ (P1)*

Not all of the survivors wanted to reconstruct the ICU-narrative. They showed avoidant behavior and were reluctant to be confronted with photographs or narratives about their time in the ICU. ICU-survivors who lacked memories of their ICU stay expressed no regret about this, as they imagined that such memories would likely be unpleasant.

##### 3.4.2 Family members: emotional recovery

In contrast to the survivors, family members had memories of the most challenging and stressful phase of their relative's illness. The ICU-narrative and the memories associated with it, still evoked strong emotions. As there were almost no shared memories of this period, it was difficult for the family members to discuss their experience – the fear, the stress, the anxious moments – with the ICU-survivor, which affected their emotional recovery.

*‘When he was in the hospital, I wrote everything down. And I tried to re-read it for myself once, but it honestly made me quite emotional. Then it really hits me again. And I can’t talk about it with him, because he didn’t go through it. So I usually talk with the children and with my daughter in particular, because she was the first on the scene.’ (F9)*

#### 3.5 Main theme 3: relationship dynamics

Relationship dynamics can be influenced by various factors, such as individual personality, past experiences and external stressors. The interviews revealed shifting feelings of connection and disconnection towards their partner among both ICU-survivors and family members.

##### 3.5.1 ICU-survivors: (Dis)Connection – navigating survival and belonging

The importance of feeling connected to each other was evoked by the confrontation with mortality and existential feelings. At the time of the interview, it was only just dawning on most ICU-survivors how close they had been to death. As a result, feeling connected to family became more important to them, though this was sometimes challenging. Some of the ICU-survivors felt disconnected due to the physical and cognitive limitations they experienced, making it difficult for them to regain their roles within the family system. Some of them felt that they no longer mattered or that they did not belong and their partner did not fully acknowledge this.

*‘It’s very confrontational. For my wife as well, of course, but I never hear her talk about it. She also never says to me, I’m so sorry for you that you can’t do more.’ (P4)*

##### 3.5.2 Family members: (Dis)Connection – navigating love and loss

In most family members existential feelings evoked a sense of realizing what is important in life.

*‘At some point, you arrive at a crossing of sorts. Where you know, yeah, there are some activities that I’ll have to stop doing. I want to focus more on the family circumstances. The relationship with my daughter has never been better. She comes over every day. To this day.’ (F1)*

Family members also expressed feelings of disconnection due to changes in the patient’s personality resulting from various medical causes, such as acquired brain injury after resuscitation or stroke. Changes in communication, labile emotional control and a lack of intimacy were often mentioned during the interviews.

*‘That intimacy thing, that’s the least difficult for me. It's just that a completely different woman came back. Sometimes, she can also explode and get very angry, slamming her fist on the table.’ (F2)*

#### 3.6 Main theme 4:Empathetic concern

Empathetic concern refers to the emotional response of compassion or care that arises when witnessing another person's distress or need. It reflects a motivation to alleviate the other person’s suffering and is distinct from simply understanding their emotions or sharing their feelings. One of the key aspects in this theme is the focus on the well-being of the other rather than the wellbeing of oneself. Both ICU-survivors and family members struggled with this.

##### 3.6.1 ICU-survivors: patient guilt

The subtheme patient guilt refers to the feelings of regret or guilt that patients experience about the impact their illness has on their partner or family member, especially if their illness has caused a burden on their loved ones. Most ICU-survivors expressed concern for their family members, particularly their partners, regarding their critical illness. They felt guilty for the distress they caused by being unconscious during the ICU-stay, as their loved ones navigated one of the most challenging times of their lives.

*‘Everybody who comes over for a visit asks me how it’s going. And my wife sits there, and she doesn’t get asked anything. But she also went through a whole lot. Because it’s not nothing, having to arrange everything at home, and the stress too…...’ (P7)*

##### 3.6.2 Family members: social ambivalence

Family members experienced empathetic concern in their ambivalent feelings toward social support offered by family and friends. While they deeply appreciated the support and kindness of others, they also found it exhausting and intrusive at times. Yet, they felt guilty for experiencing this ambivalence, believing they should only feel gratitude.

*‘There are also so many other things. You get a lot of visitors and you receive so many phone calls and messages. It’s fantastic, but also exhausting. I didn’t draw a line very often because, well, it's also encouraging.’ (F10)*

## 4 Discussion

This qualitative study explored how ICU-survivors and their family members experienced their health after ICU-discharge, and how this affected their family relations and -functioning.

The four shared main themes that we identified; personal autonomy, narrative reconstruction, relationship dynamics and empathetic concern, show how family members relate their own experience to the illness process of their relative. This finding is supported by the Family Systems Framework (Kerr and Bowen, 1988; Wright and Leahy, 2009) where one of the main principles is that illness (and care) within a family always affects everyone in the system (Kaakinen et al., 2014). The fact that the main themes manifest differently in ICU-survivors and family members, highlights the distinct nature of each of their experiences.

ICU-survivors often describe absent, severely disrupted, or distorted memories (Granja et al., 2005; Gravante et al., 2024; Lobo-Valbuena et al., 2023). Some of them may require help in reconstructing the narrative of the ICU-stay and align it with the narrative of their family members. This may help the both of them in their emotional recovery. Narrative reconstruction is a concept for understanding how people cope with changes in memory or identity and how they can adapt their personal narratives to regain a sense of meaning and coherence. Pivotal aspects of the ICU-experience are the difference in memories of ICU-survivors and their family members (Page, 2015; Page et al., 2019) and the differences they experience during the critical illness episode (Gravante et al., 2024). Storytelling enables individuals to make sense of their experiences and can help shape their personality and identity after illness or traumatic events (Frank, 2013; Schenker et al., 2015; Teece and Baker, 2017) and it may serve as an effective tool for intertwining the individual narratives of ICU-survivors and their family members. ICU diaries play an important role, as they have proven to be helpful in the emotional recovery after critical illness (Renner, 2023; Teece and Baker, 2017). However, our study shows that not all ICU-survivors feel the need to reconstruct and reconcile their ICU narrative with that of their family members, and the reverse may also be true. It is therefore important for healthcare providers to address this topic in aftercare trajectories and to provide personalized care with due regard to the family system.

The concept empathetic concern refers to an emotional state that focuses on another person, and that is triggered by and aligned with the perceived well-being of someone else (Batson, 2023). In general, little is known about patients feeling guilty for being a burden to their relatives. This phenomenon has also been described as ‘burdening guilt’ (Leonardi, 2023) and has been observed in chronically ill patients, patients with cancer and patients after a living donor procedure (Cuneoet al., 2023; Ralphet al., 2017; Saracino et al., 2023). The phenomenon of patient guilt or burdening guilt has not yet been described in the context of ICU-care. Nevertheless, the findings of our study show that it is an unexplored area in ICU-healthcare that warrants greater attention and exploration.

In the concept of Post Intensive Care Syndrome-Family, cognitive impairments are not recognized as they are in Post Intensive Care Syndrome. The cognitive impairments that several family members in our study experienced, such as difficulty concentrating and becoming easily overstimulated, may in fact be related to Post Traumatic Stress Disorder (First, 2024). Nevertheless, these impairments present a significant challenge for family members and it may be beneficial to describe these symptoms as one of the domains of Post Intensive Care Syndrome-Family, particularly in the context of providing support to family members during the ICU stay and potentially also through primary care services after hospital discharge. Rousseau et al. (2021) advocates for a modification of the PICS concept to encompass a broader framework that also integrates the consequences of the symptoms within the PICS domains. This may be beneficial for the concept of Post Intensive Care Syndrome-Family in the future.

Critical illness including an ICU-hospitalization initiates processes within the family system that can be captured in nursing diagnoses such as ‘Ineffective family health management’, ‘Impaired Family Process’, ‘Disrupted family interaction patterns’ and ‘Maladaptive family coping’, which are included in the NANDA classification (Herdman et al., 2024) (domains 1, 7 and 9). However, by incorporating some new concepts or adjusting some of the concepts in the NANDA classification, the NIC (Wagner et al., 2023), and the NOC (Moorhead et al., 2023) systems, the field of nursing could better address the holistic needs of families, leading to more comprehensive and effective care. NANDA could benefit from more contextualized diagnoses that specifically address family dynamics, roles, and interactions, such as diagnoses that capture family stress, communication issues, or the impact of a family member’s illness on the whole family. NIC could benefit from adjusting some of the interventions, designed to support the family as a whole or by adding new interventions. For example, interventions aimed at enhancing the family communication (Broekema et al., 2021; Shajani and Snell, 2023), interventions that assist family members in finding patient health promotion in daily activities, interventions to encourage regaining each individual’s personal autonomy within the family system, and interventions to help the family finding strategies to adapt to a new situation (Huber et al., 2011). The NOC system could include more outcomes that measure family wellbeing, such as improved family coping, enhanced family functioning, or reduced family stress. These outcomes will be helpful in evaluating the effectiveness of family-centered care. The current approach to post-ICU care in the Netherlands comprises a large variety of interventions, with a multi-disciplinary approach, in which ICU nurses play an important role (Schol et al., 2025). Considering the complex context of ICU aftercare, the holistic perspective that guides nursing practice appears to be an important contribution. The NANDA, NIC and NOC systems can be integrated into the Electronic Patient Record, making the use of standardized nursing terminology available during the ICU stay as well as in outpatient clinics.

Our study shows that acutely admitted ICU-survivors and patients following organ transplantation differ in their perception of their Health Related Quality of Life. Patients who underwent organ transplant are, unlike acutely admitted patients, thoroughly prepared for surgery and subsequent ICU-stay and are being offered well-structured aftercare both in-hospital and at outpatient clinics. This may position them more like elective admitted patients. Geense et al. (2021) found that 60 % of elective patients reported no physical, mental, or cognitive problems one year post-ICU, compared to 36 % of urgent surgical patients and 42 % of medical patients. Similarly, Porter et al. (2024) reported slightly higher Health Related Quality of Life in elective versus acutely admitted patients. Additionally, the pre-transplant illness and its limitations likely shape patients’ perception of the rehabilitation process, underscoring the need to consider pre-ICU status in both care settings and research.

### 4.1 Implications for clinical practice, nursing theory and future research

To date, there has been limited attention to the family system as a whole, both in ICU-care and research. Our findings indicate that an ICU-hospitalization affects the dyadic family system and likely, the entire family system on the short term. Family members may benefit from support in maintaining boundaries regarding their roles as caregivers, managing their own mental and cognitive limitations, and the family may need support in adapting to a new onset reality. The NANDA, NIC and NOC systems can be helpful in addressing family items into the ICU context and known family interventions, such as the family health conversation (Broekema et al., 2021) and the ICU diary (Renner et al., 2023).

The increasing reliance on family members as informal caregivers, due to the growing scarcity of resources (such as staff), and growing complexity in healthcare, makes it necessary to improve family-care surrounding an ICU-stay. It is therefore essential to prioritize the family system as the unit of care in both education and research. Further research to evaluate family dynamics and family interventions in the context of an ICU-hospitalization will be of added value.

### 4.2 Strengths and limitations

A strength of this study is that it has a multi-dimensional approach with ICU-nurses involved who performed the interviews. ICU-nurses are generally experienced in communicating with family members, as this is a key part of their daily activities. This helped creating a safe environment, which was supported by conducting the interviews in participants’ homes.

This study has several limitations. The RAND-36 questionnaire is developed to quantify patient perceptions about health status and QoL and we translated it into an interview guide to collect qualitative data. Although the interview questions were designed to be open-ended and as broad as possible, the RAND-36 is not meant to be used as such, which may have influenced the findings. Furthermore, the heterogeneity of the participants could be seen as a limitation. Although the participants went through the same experience, perceptions of patients following transplantation did not align with perceptions of acutely admitted patients. However, this may also be considered a strength instead of a limitation, as the ICU population is a heterogeneous population and our study may therefore be representative. Another limitation is that we interviewed only partners of ICU-survivors in this study, but an ICU-stay will also affect the rest of the family system. Finally, this study was conducted in one ICU in the northern Netherlands, which may influence the findings, as they could differ in settings with different cultures of care, family support meetings, and other family focused interventions.

## 5 Conclusions

The findings of this study provide an in-depth understanding of the experienced health of ICU-survivors and their family members. This study also highlights the need for healthcare professionals to be aware of the contrasting journey’s ICU-survivors and their family members experience and the relational dynamics this may trigger. Prioritizing attention for the family system, in care and research, is necessary to adequately organize a care path for ICU-survivors. Healthcare professionals can use the findings of this study to enhance the current approach of care after ICU discharge in any way, by actively involving the family system. Integrating approaches in diagnostic, intervention and outcome classification systems for nurses as well as in clinical practice that prioritize personal autonomy, narrative reconstruction, relationship dynamics and empathetic concern can enhance holistic patient care and support. Classification systems for nurses can be helpful in addressing family items into the ICU context and known family interventions, such as the family health conversation and the ICU diary.

## Declaration of generative AI and AI-assisted technologies in the writing process

During the preparation of this work the authors used ChatGPT in order to improve the readability and language of the manuscript. After using this tool, the authors reviewed and edited the content as needed and take full responsibility for the content of the published article.

## Funding

This research did not receive any specific grant from funding agencies in the public, commercial, or not-for-profit sectors.

## CRediT authorship contribution statement

**Marisa Onrust:** Writing – original draft, Visualization, Validation, Project administration, Investigation, Formal analysis, Data curation, Conceptualization. **Ingeborg van der Meulen:** Writing – review & editing, Validation, Supervision, Methodology, Formal analysis, Data curation, Conceptualization. **Marie Louise Luttik:** Writing – review & editing, Visualization, Validation, Supervision, Methodology, Formal analysis. **Wolter Paans:** Writing – review & editing, Visualization, Validation, Supervision, Formal analysis. **Peter H.J. van der Voort:** Writing – review & editing, Supervision. **Fredrike Blokzijl:** Writing – review & editing, Visualization, Validation, Supervision, Formal analysis, Conceptualization.

## Declaration of competing interest

The authors declare that they have no known competing financial interests or personal relationships that could have appeared to influence the work reported in this paper.

## Appendix Supplementary materials

Image, application 1
