# Supplementary material for: Health perspectives after intensive care unit-discharge: Insights from patient and family interviews
Source: Int J Nurs Stud Adv. 2025 Nov 15;10:100457. doi: 10.1016/j.ijnsa.2025.100457 (PMC12686646; doi:10.1016/j.ijnsa.2025.100457)
Supplement: Supplementary file 1 [file mmc1.zip › suppl file_ table S1_Interviewguide related to RAND-36.docx]

**Table S1. Questions interview guide related to the RAND-36**

| Questions interviewguide | | Rand-36 items |
| --- | --- | --- |
| 1 | **How do you experience your current health? [physical, emotional, social]**  *Additional questions :*   - *How would you describe your overall health? What do you think contributes to that? [feeling alive, energetic or tired or in pain]* - *Are you experiencing any physical symptoms that affect your daily life, and can you tell me more about them?* - *How do you deal with emotional distress, if you experience any? [feeling depressed or anxious]*   [If needed offer examples from the RAND-36 questionnaire to support the dialogue] | **General health** (questions 1, 33, 34, 35, 36)  **Physical functioning** (questions 3, 4, 5, 6, 7, 8, 9, 10, 11, 12)  **Emotional well-being** (questions 24, 25, 26, 28, 30)  **Social functioning** (questions 20 and 32)  **Pain** (questions 21, 22)  **Energy/fatigue** (questions 23, 27, 29, 31) |
| 2 | **What does health mean to you in your daily life?**  [If needed give examples such as in work, social life, relationships and hobbies] | **Role limitations** due to physical health  **Role limitations** due to emotional problems  **Social functioning** |
| 3 | **In what way does your current health state affect your daily life?**  *Additional questions :*   - *Would you say that it affects your family life, your work and /or hobbies?*   [If needed give examples such as work, social life, relationships and hobbies] | **Role limitations** due to physical health  **Role limitations** due to emotional problems  **Social functioning**  **Energy/fatigue** |
| 4 | **In what way has your current health state changed your daily life compared to before the ICU-stay (of your relative)?**  *Additional question :*   - *Has your (relatives’) ICU stay led to any changes in your social or societal functioning, and could you elaborate on that?* | **Health change**  **Social functioning** |
| 5 | **How do you deal with these changes?**  [Explore ways of coping, adaptation and think about family roles] | **Health change**  **Role limitations** due to physical health  **Role limitations** due to emotional problems |
| To encourage participants to eleborate on the topic:   - Could you tell me more about that? - How does that feel for you? - What makes that so important to you? | | |
