# Supplementary material for: Health perspectives after intensive care unit-discharge: Insights from patient and family interviews
Source: Int J Nurs Stud Adv. 2025 Nov 15;10:100457. doi: 10.1016/j.ijnsa.2025.100457 (PMC12686646; doi:10.1016/j.ijnsa.2025.100457)
Supplement: Supplementary file 1 [file mmc1.zip › Suppl file_tabel S2_coding scheme.docx]

**Table S2. Coding scheme with examples of meaning units and open codes**

| Examples of meaning units | Examples of open codes | Themes | Main themes |
| --- | --- | --- | --- |
| ‘ But I’m training’  ‘I must get stronger’  ‘I try to ride my bicycle again’ | Recovery | Survivor:  Physical improvement | Personal autonomy |
|  | Physical progress |  |  |
|  | Rehabilitation |  |  |
|  | Activities |  |  |
| ‘If I go shopping, I take him with me. He needs to exercise!’ | Rehabilitation of relative | Family member:  Collaborative rehabilitation |  |
|  | Joint activities to help recovery |  |  |
|  | Future perspective |  |  |
|  | Being an informal caregiver |  |  |
| ‘I remember a string of nightmares’ | Memories of frightening dreams | Survivor:  Fragmented recollections | Narrative reconstruction |
|  | No memories of ICU stay |  |  |
| ‘He didn’t go through it’  ‘My daughter understands’ | No shared experience with partner | Family member:  Emotional recovery |  |
|  | Searching for others to process the experience with |  |  |
| ‘I might not have made it, I was lucky’  ‘I’m just part of the background these days’ | Awareness of mortality | Survivor  (Dis)  Connection  Navigating survival and belonging | Relationship dynamics |
|  | Feeling irrelevant |  |  |
| ‘But I’m happy he’s still here’  ‘He has really changed, he’s much more irritable’ | Sense of finitude | Family member  (Dis)  Connection  Navigating love and loss |  |
|  | Change |  |  |
| ‘It’s also a lot what she went through’  ‘She’s afraid to come with me when I must visit the hospital’ | Worries about partner | Survivor:  Patient guilt | Empathetic concern |
|  | Causing burden |  |  |
| ‘What made it harder was how some people really insisted... and I was like, *I don’t even want to think about that right now*’ | Social support | Family member:  Social ambivalence |  |
|  | Expectations from others |  |  |
