# Supplementary material for: Health perspectives after intensive care unit-discharge: Insights from patient and family interviews
Source: Int J Nurs Stud Adv. 2025 Nov 15;10:100457. doi: 10.1016/j.ijnsa.2025.100457 (PMC12686646; doi:10.1016/j.ijnsa.2025.100457)
Supplement: Supplementary file 1 [file mmc1.zip › Supplementary file_Health persp_clean version.docx]

**Supplementary Appendix**

**Health Perspectives after Intensive Care Unit-Discharge: Insights from Patient and Family Interviews**

Marisa Onrust, MSc, RN; Ingeborg van der Meulen, PhD; Marie Louise Luttik, PhD; Wolter Paans, PhD; Peter H.J. van der Voort, MD, PhD; Fredrike Blokzijl, PhD

**Corresponding author**:

Marisa Onrust, University Medical Center Groningen, Department of Critical Care, Hanzeplein 1, 9713 GZ Groningen, The Netherlands. Email: [m.onrust@umcg.nl](mailto:m.onrust@umcg.nl). ORCID: 0000-0003-3875-373X

**Table of contents**

1. **Researcher expertise and positionality** 2
2. **Author contributions** 3
3. **Specifications of the interview guide**
   1. Table S1 Interview guide related to RAND-36 4
4. **Details of the analysis process**
   1. Figure S1 Visualization of the process of analysis 6
   2. Table S2 Coding scheme 7
5. **Data statement** 8
6. **Researcher expertise and positionality**

| Authors |
| --- |
| Marisa Onrust  Registered nurse and PhD candidate, with extensive experience in ICU care, organizing ICU research, and some experience in qualitative research. |
| Ingeborg van der Meulen  Assistant professor and lecturer in nursing research, experienced in both quantitative and qualitative research. |
| Marie Louise Luttik  Associate professor (University of Applied Sciences) with extensive expertise in Family Care and qualitative research methods. |
| Wolter Paans  Associate professor (University of Applied Sciences) with extensive expertise in nursing diagnostics and qualitative research methods. |
| Peter HJ van der Voort  Full professor with expertise in critical care, healthcare management, quality management and business research methods. |
| Fredrike Blokzijl  Assistant professor and nurse specialist, experienced in cardiothoracic care and research and ICU care and research. |
| Gratefully acknowledged |
| Renate de Beer, Willemijn Gordijn, Marjon Jalving, Linda Nieborg  All four are registered ICU nurses, with over four years of working experience in the ICU, conducting a research project as part of educational training. None of the ICU nurses had a therapeutic relationship with the participants, as none of them had cared for any of the participants during their ICU stay.  Theo Smits  Former ICU nurse and experienced research nurse, with extensive expertise in telephone interviewing. |

1. **Author contributions (CRediT)**

| Marisa Onrust | Conceptualization, Methodology, Validation, Formal analysis, Investigation, Resources, Writing – original draft, Visualization, Project administration |
| --- | --- |
| Ingeborg van der Meulen | Conceptualization, Methodology, Validation, Formal analysis, Investigation, Writing – review & editing, Supervision |
| Marie Louise Luttik | Validation, Formal analysis, Writing – review & editing, Visualization, Supervision |
| Wolter Paans | Validation, Formal analysis, Writing – review & editing, Visualization |
| Peter H.J. van der Voort | Validation, Writing – review & editing, Visualization, Supervision |
|  |  |
| Fredrike Blokzijl | Validation, Formal analysis, Writing – review & editing, Visualization, Project administration |

1. **Specifications of the interview guide**

The table below provides detailed information on how the questions in the interview guide are related to the RAND-36 questionnaire.

**Table S1. Questions interview guide related to the RAND-36 questionnaire**

| Questions interview guide | | Rand-36 items |
| --- | --- | --- |
| 1 | **How do you experience your current health? [physical, emotional, social]**  *Additional questions :*   - *How would you describe your overall health? What do you think contributes to that? [feeling alive, energetic or tired or in pain]* - *Are you experiencing any physical symptoms that affect your daily life, and can you tell me more about them?* - *How do you deal with emotional distress, if you experience any? [feeling depressed or anxious]*   [If needed offer examples from the RAND-36 questionnaire to support the dialogue] | **General health** (questions 1, 33, 34, 35, 36)  **Physical functioning** (questions 3, 4, 5, 6, 7, 8, 9, 10, 11, 12)  **Emotional well-being** (questions 24, 25, 26, 28, 30)  **Social functioning** (questions 20 and 32)  **Pain** (questions 21, 22)  **Energy/fatigue** (questions 23, 27, 29, 31) |
| 2 | **What does health mean to you in your daily life?**  [If needed give examples such as in work, social life, relationships and hobbies] | **Role limitations** due to physical health  **Role limitations** due to emotional problems  **Social functioning** |
| 3 | **In what way does your current health state affect your daily life?**  *Additional questions :*   - *Would you say that it affects your family life, your work and /or hobbies?*   [If needed give examples such as work, social life, relationships and hobbies] | **Role limitations** due to physical health  **Role limitations** due to emotional problems  **Social functioning**  **Energy/fatigue** |
| 4 | **In what way has your current health state changed your daily life compared to before the ICU-stay (of your relative)?**  *Additional question :*   - *Has your (relatives’) ICU stay led to any changes in your social or societal functioning, and could you elaborate on that?* | **Health change**  **Social functioning** |
| 5 | **How do you deal with these changes?**  [Explore ways of coping, adaptation and think about family roles] | **Health change**  **Role limitations** due to physical health  **Role limitations** due to emotional problems |
| To encourage participants to eleborate on the topic:   - Could you tell me more about that? - How does that feel for you? - What makes that so important to you? | | |

1. **Details of the analysis process**

Below a schematic overview is shown with details about the full research process step by step. At the end of phase 1 the interview guide was adapted based on the fieldnotes and observations. At the end of phase 2 data saturation was confirmed by employing two extra interviews. In phase 3, all collected data were consolidated by the researchteam (all authors), and a re-analysis of all audio recordings and transcripts was conducted. Finally, a visualization of the main themes and themes was made.


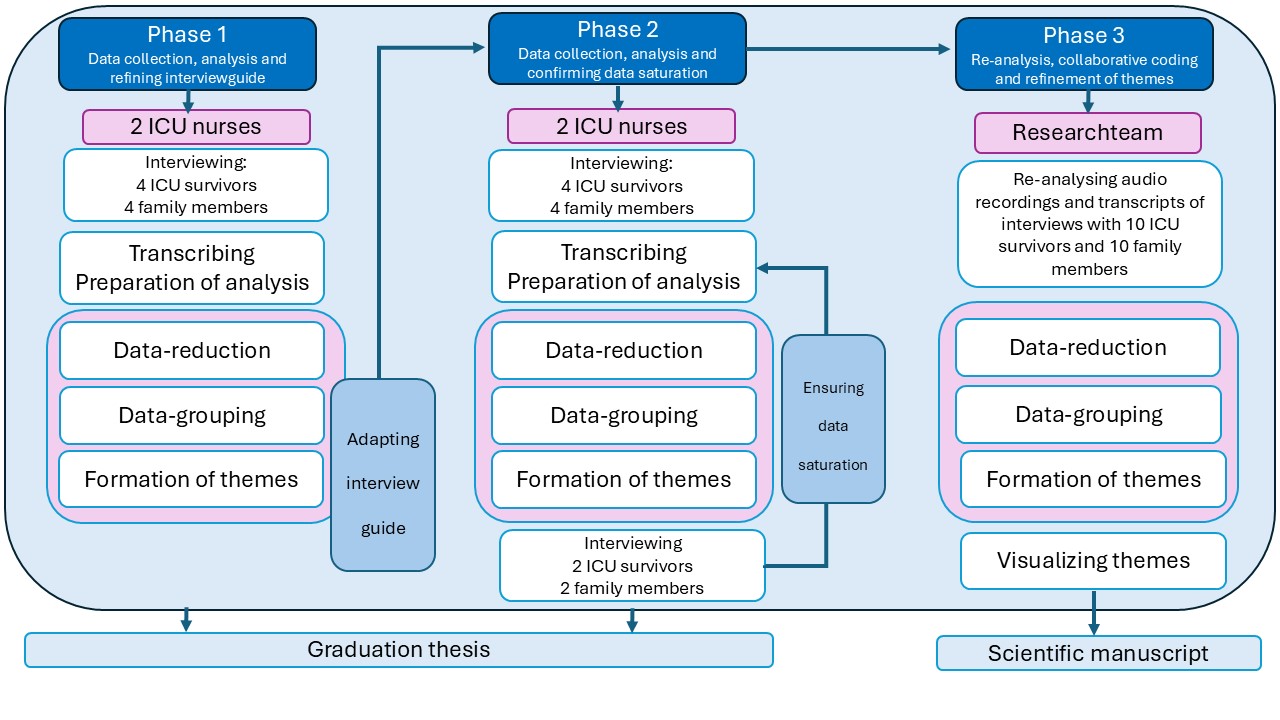
**Figure S1. Visualization of the process of inductive content analysis**

**Table S2. Coding scheme with examples of meaning units and open codes**

| Examples of meaning units | Examples of open codes | Themes | Main themes |
| --- | --- | --- | --- |
| ‘ But I’m training’  ‘I must get stronger’  ‘I try to ride my bicycle again’ | Recovery | **Survivor:**  Physical improvement | **Personal autonomy** |
|  | Physical progress |  |  |
|  | Rehabilitation |  |  |
|  | Activities |  |  |
| ‘If I go shopping, I take him with me. He needs to exercise!’ | Rehabilitation of relative | **Family member:**  Collaborative rehabilitation |  |
|  | Joint activities to help recovery |  |  |
|  | Future perspective |  |  |
|  | Being an informal caregiver |  |  |
| ‘I remember a string of nightmares’ | Memories of frightening dreams | **Survivor:**  Fragmented recollections | **Narrative reconstruction** |
|  | No memories of ICU stay |  |  |
| ‘He didn’t go through it’  ‘My daughter understands’ | No shared experience with partner | **Family member:**  Emotional recovery |  |
|  | Searching for others to process the experience with |  |  |
| ‘I might not have made it, I was lucky’  ‘I’m just part of the background these days’ | Awareness of mortality | **Survivor**  (Dis)  Connection  Navigating survival and belonging | **Relationship dynamics** |
|  | Feeling irrelevant |  |  |
| ‘But I’m happy he’s still here’  ‘He has really changed, he’s much more irritable’ | Sense of finitude | **Family member**  (Dis)  Connection  Navigating love and loss |  |
|  | Change |  |  |
| ‘It’s also a lot what she went through’  ‘She’s afraid to come with me when I must visit the hospital’ | Worries about partner | **Survivor:**  Patient guilt | **Empathetic concern** |
|  | Causing burden |  |  |
| ‘What made it harder was how some people really insisted... and I was like, *I don’t even want to think about that right now*’ | Social support | **Family member:**  Social ambivalence |  |
|  | Expectations from others |  |  |

1. **Data statement**

*Research data for this article*

Due to the sensitive and personal nature of the data collected in this study, participants did not consent to the sharing of raw interview transcripts. Therefore, the full dataset is not publicly available. However, summarized or anonymized excerpts may be available upon reasonable request from the corresponding author, subject to ethical approval.
