# Supplementary figures and images for: Health perspectives after intensive care unit-discharge: Insights from patient and family interviews
Source: Int J Nurs Stud Adv. 2025 Nov 15;10:100457. doi: 10.1016/j.ijnsa.2025.100457 (PMC12686646; doi:10.1016/j.ijnsa.2025.100457)

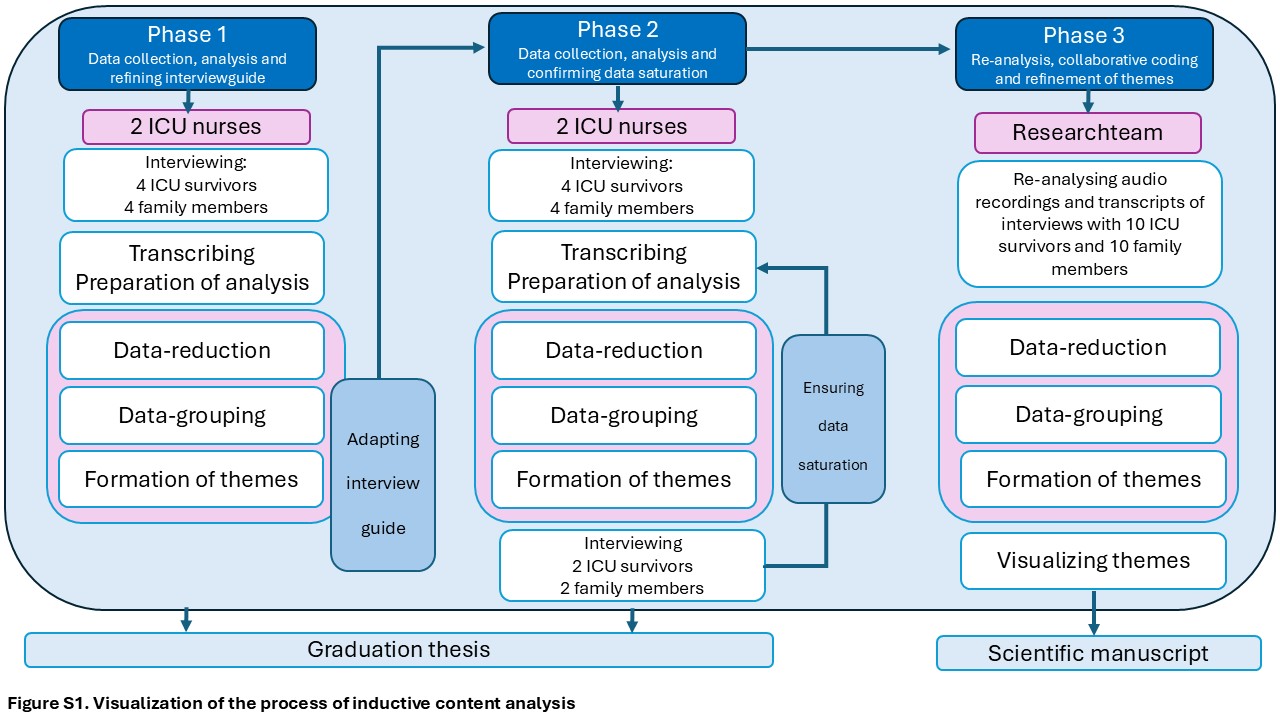

Supplement: Supplementary file 1 [file mmc1.zip › Suppl file_fig.S1-visual of contentanalysis_v1.1_caption.jpg]

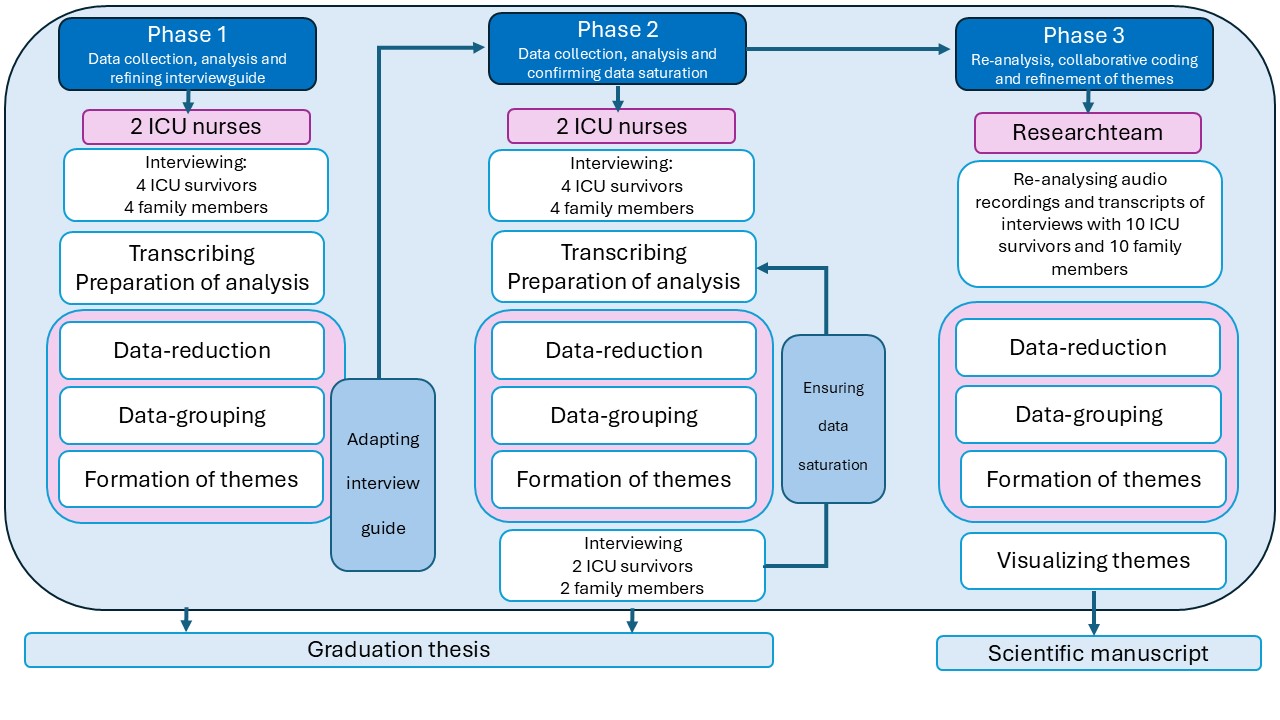

Supplement: Supplementary file 1 [file mmc1.zip › Suppl file_fig.S1-visual of contentanalysis_v1.1_NO capt.jpg]
